# Occupational Risk Factors by Sectors: An Observational Study of 20,000 Workers

**DOI:** 10.3390/ijerph20043632

**Published:** 2023-02-18

**Authors:** Luther Dogbla, Cédric Gouvenelle, Florence Thorin, François-Xavier Lesage, Marek Zak, Ukadike Chris Ugbolue, Barbara Charbotel, Julien S. Baker, Bruno Pereira, Frédéric Dutheil

**Affiliations:** 1 Physiological and Psychosocial Stress, CNRS UMR 6024, LaPSCo, University Clermont Auvergne, CHU Clermont-Ferrand, Occupational Medicine, 63000 Clermont-Ferrand, France; 2 Occupational Health Service of Cher (APST18), 18000 Bourges, France; 3 CNRS, Acté, University Clermont Auvergne, 63000 Clermont-Ferrand, France; 4 IDESP, INSERM, CHU Montpellier, University Montpellier, 34000 Montpellier, France; 5 Faculty of Medicine and Health Sciences, Institute of Physiotherapy, The Jan Kochanowski University, 25-369 Kielce, Poland; 6 School of Health and Life Sciences, Institute for Clinical Exercise & Health Science, University of the West of Scotland, Glasgow G72 0LH, UK; 7 UMRESTTE, Domaine Rockefeller, University Lyon 1, 69373 Lyon, France; 8 Centre for Health and Exercise Science Research, Hong Kong Baptist University, Hong Kong 999077, China; 9 Clinical Research and Innovation Direction, CHU Clermont-Ferrand, 63000 Clermont-Ferrand, France; 10 Physiological and Psychosocial Stress, CNRS UMR 6024, LaPSCo, University Clermont Auvergne, CHU Clermont-Ferrand, Occupational Medicine, WittyFit, 63001 Clermont-Ferrand, France

**Keywords:** occupational health, work exposure, sectors of activity, prevalence

## Abstract

Objective: We aimed to assess the prevalence of exposure by sector and the sectors of activity most exposed to each exposure, using routine occupational health data, and to quantify the risk of being exposed. Method: Occupational risk factors were assessed by workers followed by the Occupational Health Service of Cher, using self-reported questionnaires. The sectors of activity were grouped into seven sectors, and the risks were grouped into six occupational exposure groups. Comparisons were made using the Chi-squared test and Cramer’s V, and the odds ratios were calculated by using logistic regression. Results: We included 19,891 workers. The construction sector had the highest prevalence (*p* < 0.05 vs. all other sectors) of exposure to physical (76%) and biomechanical factors (82%), as well as chemical risks (75%). Human health and social work was the sector with the highest prevalence of exposure to biological factors (69%), psychosocial factors (90%), and atypical working hours (61%). With workers from administrative and support sectors as the reference, construction workers had more chance of declaring exposure to physical factors (OR = 3.28, 95%CI = 2.89 to 3.72), biomechanical factors (1.82, 1.58 to 2.09), and chemical agents (3.83, 3.38 to 4.33). Workers from the human health and social sectors had more chance of being exposed to biological agents (13.4, 11.9 to 15.2), atypical working hours (1.93, 1.75 to 2.14), and psychosocial factors (2.74, 2.38 to 3.16). Conclusion: Psychosocial risk factors were commonly reported in all sectors. Workers in the construction, human health, and social sectors seem to report more exposures than those in other sectors. The analysis of occupational exposures is a necessary basis to build an efficient preventive strategy for occupational health.

## 1. Introduction

Workers, in the context of their professional activities, are in contact with some occupational risks. Exposure to occupational risk factors is a major public health issue, and also generates a huge economic impact for companies [1]. An inventory of those occupational risk factors is needed to build an efficient preventive strategy [2]. These risk factors can be exposure to some agents (chemical, biological, biomechanical, or physical) or constraints, either organizational or relational. These are families of exposures, within which there are exposures of the same type or with similar consequences for the workers. The type of exposure depends on the sector of activity [3,4,5]; however, the available studies have mostly focused on the prevalence of exposure to different risks in some specific sectors [6,7,8]. For example, the construction and manufacturing sector have higher levels of exposure to physical agents such as noise and vibration compared with other sectors [9,10]. Other studies on human health and social workers have reported the common exposure to biological agents as a result of being in contact with humans or human products at work [11,12]. There are some national epidemiological surveys, such as the SUMER study (Surveillance Médicale des Expositions aux Risques Professionnels, i.e., Medical Monitoring of Occupational Risk Exposure), the EVREST observation (Évolutions et Relations en Santé au Travail i.e., Occupational Health Evolution and Relations), and the NHANES (National Health And Nutrition Examination Survey), which also collect occupational exposures [13,14]. However, to our knowledge, no study has provided an overview of all occupational exposures by sector. Additionally, some studies have focused on the sociodemographic profiles of workers exposed to a very specific risk from a dedicated sector [15,16,17]. It may be relevant to know the exposures attached to each sector of activity, independently of the profiles of the workers. There is a need for occupational physicians, as well as for company directors or employees, to have easy access to all the risks of each sector. Very interestingly, occupational health software now makes it possible to obtain quality mass data in real life, outside of the context of a specific study [18]. In addition, occupational health services today rely on standard thesauri to list and classify work situations into occupational exposure families.

Therefore, the main objective of this study was to assess the prevalence of exposure by sector and the sectors of activity most exposed to each type of exposure, using routine occupational health data. The secondary objective was to quantify the risk of being exposed to each occupational risk factor depending on the sector.

## 2. Materials and Methods

### 2.1. Study Design

**Figure 1:**
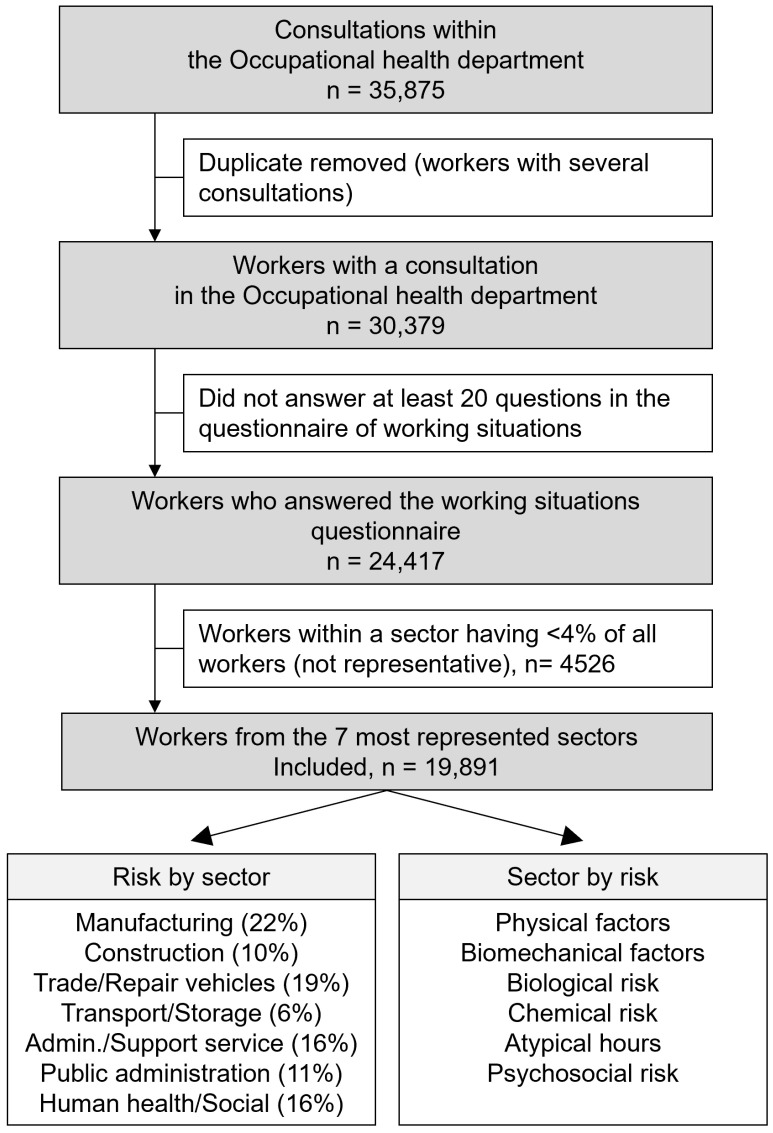
Flow chart of the data collection process.

We carried out a cross-sectional study on the exposure declarations of workers followed by the occupational health service known as the Association de Prévention en Santé au Travail du Cher (APST18). Occupational risk factors were assessed using self-report questionnaires that were proposed systematically to workers followed by the APST18. Questionnaires were proposed before each occupational health consultation. The study covered all workers seen for a medical checkup between 1 January 2021 and 31 December 2021. Padoa software was used to implement the questionnaire, and the data were extracted anonymously (Figure 1).

### 2.2. Study Sample 

The inclusion criteria for workers were to have had a medical checkup during the study period, and to have answered at least 20 questions in the questionnaire. In the case where a worker had made several visits during the period, the visit considered was the last one where the worker had filled in the associated visit questionnaire.

### 2.3. Occupational Risk Factors

Workers had to choose occupational risk factors from a list within six families of exposure: physical, biomechanical, biological, chemical, atypical hours, and psychosocial risks. Physical factors include exposure to vibration, working in bad weather, ultrasound, radiation (ionizing or not), and extreme temperatures. Biomechanical factors include exposure to awkward postures, heavy loads, and repetitive movements. Biological risk factors are defined as living or dead biological material that may have harmful effects on humans or the environment [19]. This includes work in contact with humans and animals or their products, waste processing in the environment or in the food industry, and contact work. Exposures to chemical agents include exposure to fibers, fumes, gases, and dusts, and handling chemicals or cosmetics. Atypical hours are mostly shift/night work, weekend work, and irregular working hours. Psychosocial risk factors include aspects such as the emotional demands of the job, work schedules, autonomy of organization, anxiety related to the socio-economic situation of the company, interpersonal relationships at work, and conflict at work [6]. The details of the occupational risk factors for each category of risk are listed in Appendix A. Each exposure was retrieved as qualitative data (yes/no).

### 2.4. Sectors of Activity

The sectors of activity were coded according to the European NACE-2008 classification and the international aggregated classification A17. NACE stands for “Nomenclature statistique des activités économiques dans la Communauté européenne,” which is French for “Statistical classification of economic activities in the European Community”. This classification system allows for a standardized and widely recognized method of grouping the sectors of activity, allowing for comparability and consistency in data analyses across different studies and contexts [3,20,21]. We included only sectors that had over 500 workers (2% of the respondents). We then grouped the sectors of activity of the workers into 7 sectors: manufacturing, construction, trade and repair of motor vehicles and motorbikes, transport and warehousing, administrative and support service activities, public administration, and human health and social work. For more details on the activities related to each sector, please refer to Appendix B.

### 2.5. Statistics

**Figure 2:**
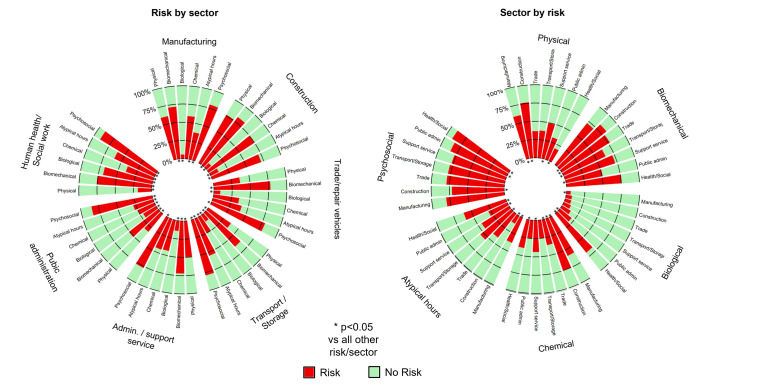
This plot presents a polar representation of the percentage of workers who declared an exposure to a certain risk. The red color represents the percentage of individuals who reported an exposure, while the grey color represents the percentage of individuals who did not report an exposure. On one side of the plot, the declarations are grouped by sector (**left**); on the other side, they are grouped by risk (**right**). * *p* < 0.05 vs. all other risks/sectors.

**Figure 3:**
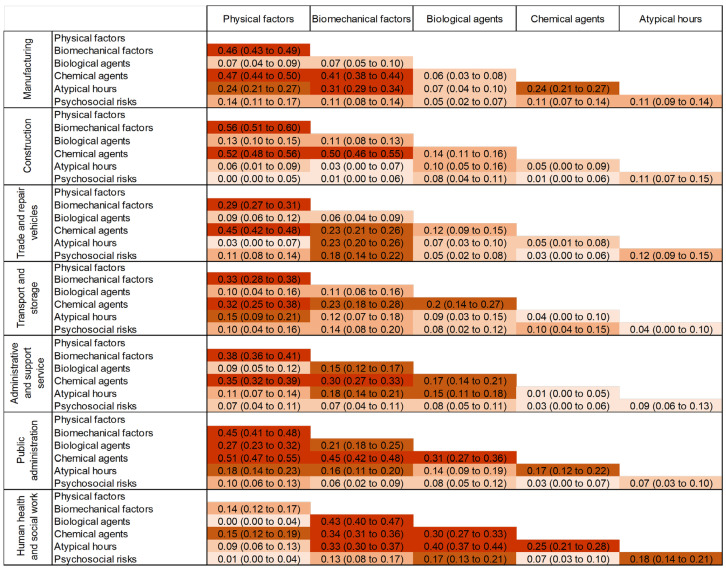
Heatmap of Cramer’s V (i.e., effect size (ES)): risk by sector.

**Figure 4:**
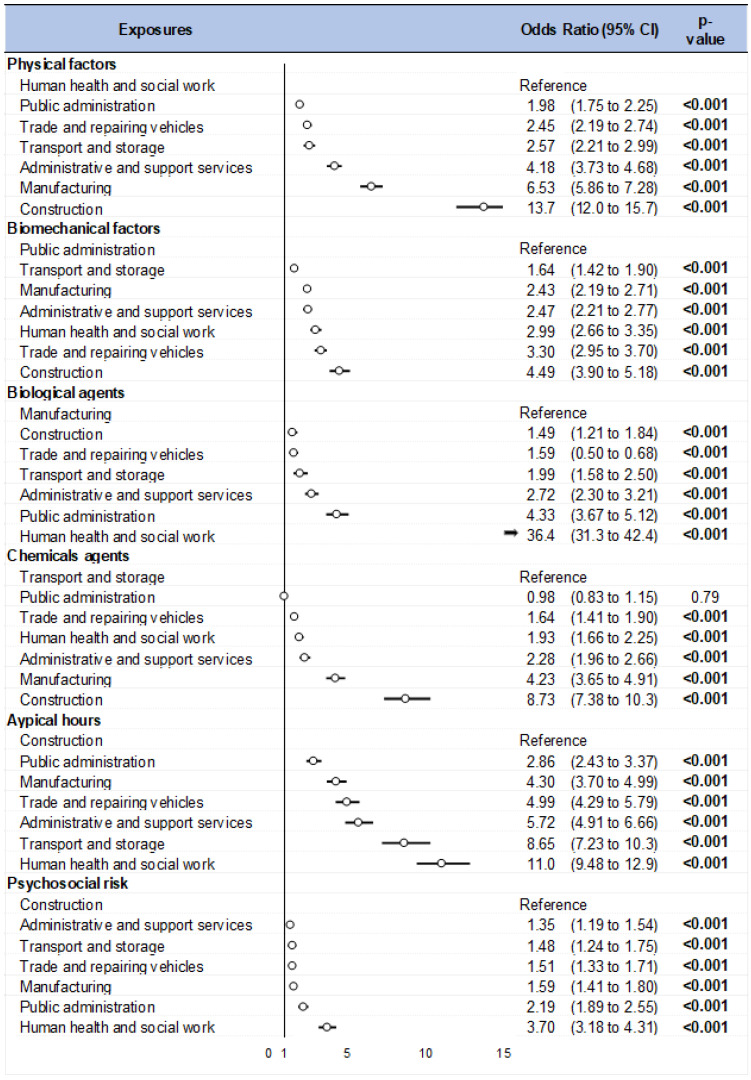
Forest plot: Sector by risk.

Note: The circle represents the point estimates for each sector and the horizontal lines indicate the confidence intervals around the point estimates. The vertical line indicates the null effect, which is the point at which the odds ratio equals one. If the confidence interval for a sector crosses the vertical line, it suggests that the odds ratio is not statistically significant. For some sectors, an arrow indicates that the point estimate is outside the range of the plot and the circle is cut off. 

Categorical data are expressed as numbers (n) and percentages (%). The Chi-squared test was used to compare variables among groups (risk by sector, and sector by risk) (Figure 2). We used Cramer’s V to measure the effect size among each of the risks and sectors (Figure 3). Cramer’s V varies between 0 and 1 without any negative values, where 0 means no association. However, a value bigger than 0.25 is considered as a very strong relationship for Cramer’s V (Appendix C). We then calculated the odds ratios of being exposed to each occupational risk factors, depending on the sector, by carrying out a logistic regression for each sector with the six occupational risk factors as explanatory variable (risk by sector). When the reference class was not clearly identifiable, it is common to use the class with the lowest prevalence, mean, or highest prevalence [19]. The reference class chosen for each exposure was the sector with the lowest reported exposure to that exposure family. The results were expressed as odds ratios (OR). Forest plots were used to present the results (Figure 4). Statistical analysis was performed using Stata software, version 14.0 (StataCorp, College Station, TX, USA). A two-sided Type I error of 5% was applied for all statistical tests. As such, a difference was considered statistically significant when *p* ≤ 0.05. Pairwise comparisons were performed between groups using Bonferroni tests when a difference between more than two groups was retrieved.

## 3. Results

In 2021, 24,456 workers (40 ± 13 years, 60.6% men) had a medical check-up and completed at least 20 questions in the worker questionnaire, i.e., approximately 81% of the workers seen at APST18 during the year 2021. The seven most represented sectors alone accounted for approximately 81% (19,891) of the workers retained. These workers were distributed as follows: manufacturing, 22%; vehicle trade and repair, 19%; administrative and support service activities, 16%; human health and social work, 16%; public administration, 11%; construction, 10%; transport and warehousing, 6% (Appendix D). The sample studied is representative of the population of workers followed by the department.

### 3.1. Prevalence of Occupational Risk Factors by Sector (Risks by Sector)

Manufacturing: The most frequent exposure was psychosocial (80%, 95 CI = 78 to 81%), followed by biomechanical factors (72%, 70 to 73%), then physical (60%, 59 to 62%) and chemical risks (59%, 57 to 60%), then atypical hours (38%, 36 to 39%) (Figure 2). Biological risks were rare (6%, 5 to 6%). All risks showed significant differences from each other, with varying degrees of strength (*p* < 0.001 for Chi^2^, ES from 0.05 to 0.46 for Cramer’s V), except for physical and chemical risks. The differences observed for biomechanical factors and chemical agents, physical factors, and atypical hours were very strong (ES > 0.25), and the relationship between physical and chemical agents was also very strong (ES = 0.47) (Figure 3).

Construction: Four risks were very frequent (>70%): biomechanical (82%, 80 to 84%), followed by physical (75%, 73 to 76%) and chemical (76%, 74 to 78%), then psychosocial risks (71%, 69 to 73%) (Figure 2). Biological risks and atypical hours were uncommon (<15%). Except for an absence of a difference between physical and chemical risks, all other risks differed from each other themselves (*p* < 0.001), despite some very tenuous differences for some comparisons (ES from 0.001 to 0.56). Very strong differences were observed for biomechanical, chemical, and physical factors (ES > 0.50) (Figure 3).

Trade and repair of motor vehicles and motorbikes: The most frequent exposures were psychosocial risks (79%, 78 to 80%) and biomechanical factors (77%, 76 to 79%). These were followed by atypical working hours (41%, 95% CI: 40 to 43%), physical factors (36%, 34 to 38%), and chemical risks (35%, 34 to 38%). Biological risks were very seldom reported (9%, 8 to 10%) (Figure 2). Only biological risks differed from all other risks, with moderate strength (*p* < 0.001, ES from 0.05 to 0.12) (Figure 3).

Transport and storage: Exposure to psychosocial factors was the most dominant exposure (79%, 76–81), followed by biomechanical factors (63%, 60 to 66%), atypical working hours (55%, 52 to 58%), physical factors (38%, 35 to 40%), chemical risks (25%, 23 to 28%), and biological risks (11%, 9 to 23%) (Figure 2). The differences in prevalence among all risks differed in their strength. (*p* < 0.001, ES from 0.04 to 0.33). The strongest differences were observed for biomechanical factors, physical factors, and chemical agents (ES > 0.15) (Figure 3). 

Administrative and support service activities: The most prevalent risks were psychosocial risks (77%, 76 to 79%) and biomechanical factors (72%, 70 to 74%). These were followed by physical factors (49%, 48 to 51%), which had no difference from reported exposure to chemical factors and atypical working hours, respectively (43% and 44%, 95% CI 42 to 56%). The least reported was biological risks (14%, 13 to 15%) (Figure 2). Strong ES values were found between biomechanical factors and all other risks (ES from 0.15 to 38), except for psychosocial factors (ES = 0.07). Chemical and physical risks also showed a very strong effect size ES = 0.35 (Figure 3).

Public administration: The highest reported risks were psychosocial risks (85%, 83 to 86%), followed by biomechanical factors (51%, 49 to 53%), then physical factors (32%, 30 to 34%), and lastly atypical working hours (29%, 27 to 30%), chemical risks (25%, 23 to 27%), and biological risks (21%, 19 to 22%) (Figure 2). Psychosocial and biomechanical factors differed from all other factors (*p* > 0.001, ES from 0.03 to 0.51). The analysis of the effect sizes showed strong relationships among all risks except for psychosocial factors (ES < 0.10) (Figure 3). 

Human health and social work: Despite a high prevalence of biological risks (69%, 67 to 70%), psychosocial factors remained the most prevalent (90%, 89 to 91%). Next were biomechanical factors (72%, 74 to 77%) and atypical working hours (61%, 59 to 62%), and the least declared were chemical risks (39%, 38 to 41%) and physical factors (19%, 18 to 20%) (Figure 2). Psychosocial, biological, and physical factors were different from all others, with different strengths (*p* < 0.001, ES from 0.00 to 0.43). The strongest differences were among biomechanical factors, biological agents, and chemical agents (ES > 25) (Figure 3).

### 3.2. Prevalence of Sectors for Each Occupational Risk Factor (Sectors by Risk)

Physical factors: The sectors with the highest prevalence are construction (76%, 74 to 78%) and manufacturing (60%, 59 to 62%), followed by administrative and support services, then transport and storage, vehicle trade and repair, and public administration (38%, 36%, and 32%, respectively). Human health and social work was the sector with the least exposure (Figure 2). All sectors were different from each other (*p* < 0.001), except for transport and storage, vehicle trade and repair, and public administration (ES = 0.01). Cramer’s V showed strong differences, with the ES being between 0.01 and 0.57. Very strong ES values were found between construction and other sectors (ES between 0.27 and 0.57) (Appendix E). 

Biomechanical factors: The highest prevalence was in construction (82%, 80 to 84%) then in human health and social work (76%, 74 to 77%), manufacturing, and administrative and support services (72%, 70 to 74%). Those with the lowest prevalence were transport and storage (63%, 60 to 66%) and public administration (51%, 49 to 53%) (Figure 2). Construction, transport and storage, and public administration were significantly different from all other sectors (*p* < 0.001, ES from 0.06 to 0.33). These differences appeared to be strong for public administration (ES = 0.33) and for transport and storage (ES = 0.22) (Appendix E). 

Biological risk: Human health and social work was by far the sector with the most workers exposed to biological risks (69%, 67 to 70%), followed by public administration with 21% (19 to 22%) then administrative and support services (14%, 13 to 15%), transport and storage (11%, 9 to 13%), vehicle trade and repair (9%, 8 to 10%), and construction and manufacturing (8% and 6%, respectively, 5 to 9%) (Figure 2). Human health and social work, and public administration were the ones that showed significant differences from all the other sectors (*p* < 0.001). Very strong differences were found for the comparisons between the human health and social work sector and the other sectors (ES between 0.47 and 0.66). Public administration also showed strong differences (ES between 0.17 and 0.47) except with transport and storage (ES = 0.12), and administrative and support services (ES = 0.09) (Appendix E).

Chemical risk: The construction sector was the most exposed (75%, 73 to 76%), followed, in order, by manufacturing (59, 57 to 60%), social and human health work (39%, 38 to 41%), trade and repair of motor vehicles (35%, 34 to 36%), and public administration, and transport and storage (25%, 23 to 28%) (Figure 2). Construction and manufacturing were significantly different from all other sectors (*p* < 0.001, ES from 0.15 to 0.50) (Appendix E). 

Atypical hours: Workers in the human health and social work sector were the most exposed to atypical working hours (61%, 59 to 62%), followed by transport and storage (55%, 52 to 58%). Vehicle trade and repair, and administrative and support services were not significatively different (41% and 44%, respectively, 40 to 56%). They were followed by manufacturing (38%, 36 to 39%), public administration (29%, 27 to 30%) and construction (12%, 11 to 14%). Except for vehicle trade and repair, and administrative and support services, all other sectors were significantly different (*p* < 0.001) (Figure 2). These differences were strong between construction and each other sector (ES from 0.20 to 0.48). The human health and social work sector showed also strong differences from each sector (ES from 0.16 to 0.48), except transport and storage (ES = 0.05) (Appendix E). 

Psychosocial risk: All sectors showed a high prevalence of reported exposure (from 71 to 90%). Human health and social work were the most exposed sectors (90%, 89 to 91%), followed by public administration (85%, 83 to 86%), manufacturing (80%, 78 to 81%), transport and storage, trade and repair of motor vehicles (79%, 76 to 81%), administrative and support services (77%, 76 to 78%), and construction (71%, 69 to 73%) (Figure 2). Construction, public administration, and human health and social work were the sectors that differed most from the others (*p* < 0.001). The analysis of the effect sizes showed generally weak differences, except for a few exceptions. Human health and social work was the only sector with strong differences from the other sectors (ES ranging from 0.15 to 0.24), except for manufacturing and public administration. The difference between construction and public administration also appeared to be strong (ES = 0.16) (Appendix E). 

### 3.3. Odds Ratio for each Sector by Risk

Using human health and social work as reference, the risk of being exposed to physical factors was multiplied by 1.98 times (95%CI: 1.75 to 2.25) for public administration and by 2.45 (2.19 to 2.74) for vehicle trade and repair. For transport and storage, the risk was multiplied by 2.57 (2.21 to 2.99), and it was multiplied by 4.18 (3.73 to 4.68) for administrative and support services. The highest odds ratios were found for manufacturing and construction: 6.53 (5.86 to 7.28) and 13.7 (12.0 to 15.7), respectively. Biomechanical factors were analyzed using public administration as the reference. The results indicated that the risk of exposure to biomechanical factors was multiplied by 1.64 (1.42 to 1.90) in the transport and storage sector, by 2.43 (2.19 to 2.71) in manufacturing, by 2.47 (2.21 to 2.77) in administrative and support services, by 2.99 (2.66 to 3.35) in human health and social work, by 3.30 (2.95 to 3.70) in vehicle trade and repair, and by 4.49 (3.90 to 5.18) in construction. Using manufacturing as the reference, the odds of being exposed to biological agents were 1.49 times higher (1.21 to 1.84) in the construction sector, 1.59 times higher (1.30 to 1.68) for vehicle trade and repair, 1.99 times higher (1.58 to 2.50) for transport and storage, 2.72 times higher (2.30 to 3.21) for administrative and support services, 4.33 times higher (3.67 to 5.12) for public administration, and 36.4 times higher (31.3 to 42.4) for human health and social work. Using transport and storage as the reference, the odds of exposure to chemical agents seemed to be the same for public administration (OR = 0.98 (0.83 to 1.15)). The odds were multiplied by 1.64 (1.41 to 1.90) for vehicle trade and repair. The risk of being exposed was multiplied by 1.93 (1.66 to 2.25) for human health and social work, 2.28 (1.96 to 2.66) for administrative and support services, and 4.23 (3.65 to 4.91) for manufacturing. The highest odds ratio was found for construction at 8.73 (7.38 to 10.3). With reference to construction, employees in public administration faced a 2.86-fold (2.43 to 3.37) increase in the risk of working atypical hours. In manufacturing, the risk was elevated by 4.30 (3.70 to 4.99). The vehicle trade and repair sector also had a 4.99-fold (4.29 to 5.79) increase in the risk. Administrative and support services faced a 5.72-fold (4.91 to 6.66) increase, while transport and storage had a higher risk, with an 8.65-fold (7.23 to 10.3) increase. The risk was the highest for human health and social work, with an 11.0-fold (9.48 to 12.9) increase. In terms of psychosocial risk, the reference category was construction. When comparing sectors, administrative and support services had a risk ratio of 1.35 (1.19 to 1.54), transport and storage had a risk ratio of 1.48 (1.24 to 1.75), vehicle trade and repair had a risk ratio of 1.51 (1.33 to 1.71), manufacturing had a risk ratio of 1.59 (1.41 to 1.80), public administration had a risk ratio of 2.19 (1.89 to 2.55), and human health and social work had the highest risk ratio of 3.70 (3.18 to 4.31).

## 4. Discussion

The main findings showed that there were differences in the prevalence of reported exposure across sectors, but that psychosocial risk factors were commonly reported in all sectors. It is also found that workers in some sectors reported more exposures than others, notably construction, and human health and social work.

### 4.1. Prevalence

Construction workers had the highest prevalence of exposure to physical factors, followed by the manufacturing sector. An analysis of the results of the SUMER 2017 survey based on occupational health declarations by professionals showed that workers working mainly outdoors were almost all exposed to factors such as severe weather and a noisy work environment, which are factors constituting the family of physical exposure [22]. Other studies on the consequences of exposure to vibration showed that these two sectors were the ones with the highest prevalence of exposure to vibration and extreme temperatures [7,23,24]. The lowest exposure to physical factors was reported by workers in the human health and social work sector. The human health and social work sector was mainly composed of hospitals, medical and social accommodation, and social action without accommodation. Workers in these sectors seldom work in an outdoor environment and use very few tools that can cause vibrations. Moreover, in these types of environments it is recommended to make as little noise as possible to preserve the health of the patient, which would explain the low level of declarations by these workers of exposure to physical factors [25]. This result is in line with the analysis that was made on a systematic review of noise exposures [26]. Workers in the construction sector had the highest prevalence of reporting biomechanical factors. This is consistent with the results found in other studies using the sector of activity as a variable [27]. In these sectors of activity, workers often must handle loads and make repetitive motions with equipment, which is sometimes not adapted to the working conditions. Public administration was the sector with the lowest reported prevalence of exposure to biomechanical factors. The low exposure to biomechanical factors among public administration workers can be attributed to the heterogeneous nature of the sector, encompassing a variety of trades with disparate duties primarily focused on legislation, in contrast to the other sectors studied, which encompassed trades characterized by numerous work situations involving biomechanical exertion [28]. Furthermore, many workers within the public administration sector engage in desk-based tasks, such as typing, writing, or reading, which reduce the physical demands placed on the body. Work situations exposed to biomechanical factors are known to play a role in the appearance of musculoskeletal disorders [29], which are the main sources of work-related morbidity [30], and in the loss of productivity [31,32]. This study supports the importance of controlling these exposures through the installation of suitable equipment and tools, taking action in the workplace, and adapting the workstation if necessary [33]. The human health and social work sector was the sector with the highest prevalence of reported exposure to biological agents. This excess of exposure compared with other sectors has been discussed in several studies [11,12], including a study published in 2014 on work-related chemical and biological risks in all occupations in Europe [34]. The study explained this particularity of this sector by, on the one hand, elements specific to the sector (the environmental and social context, tasks etc.) and, on the other hand, by the characteristics of biological agents (means of exposure, pathogenicity, transmission mechanisms, etc.). Exposure to chemical agents was reported mainly by workers in the construction and manufacturing sectors. Workers in the manufacturing sector are known to have high exposure to chemical agents [35]. Workers in these two sectors handle and are exposed to chemicals in their daily work, either through dust emissions or through the chemicals used to perform their tasks. Construction workers may be exposed to chemicals agents through a variety of activities on the job site. These include handling hazardous materials such as adhesives, solvents, and cleaning agents, as well as exposure to fumes, gases, dust, and fibers from building materials such as asbestos, etc. For instance, a construction worker may be involved in mixing and applying chemicals to treat concrete surfaces, or in sanding and cutting materials that generate fumes and dust [36]. Data have shown that workers in both the construction and manufacturing sectors were primarily exposed to chemicals or cosmetics, as well as fumes, gases, and dust. However, it was observed that workers in the manufacturing sector reported more frequent handling of chemicals or cosmetics compared with those in the construction sector. On the other hand, construction workers reported a higher frequency of exposure to fumes, gases, and dust. Exposure to atypical working hours is very common in the hospital sector and in the transport and storage sector [37]. These workers most often combine shift work, night work, and on-call duty. Even in France, legislation offers more latitude in the number of night hours that can be worked in the public hospital sector than in the private sector. In the hospital setting or in certain industries, these atypical hours are more because the service is obliged to be continuous, which is a constraint specific to the sector. Exposure to psychosocial factors concerns all types of workers [38], regardless of their sector, which explains why it is the exposure group with the highest levels of exposure across all sectors. However, workers in the health sector are known to have high levels of exposure to psychosocial risk factors [16]. Compared with workers in other sectors, workers in the health sector were found to have higher reported exposures [39].

### 4.2. Odds Ratios 

Workers in the construction sector were, unsurprisingly, the most likely to report exposure to the family of physical factors, followed by the manufacturing sector. This is understandable because these workers are often outdoors, which exposes them to the various factors that make up this family of exposures [40]. The other sectors, therefore, appeared to be protective against exposure to physical factors. This is an argument in favor of paying particular attention to workers in the construction and manufacturing sectors regarding the importance of protective measures against these factors. The construction sector had also the highest risk compared with the administrative and service support sectors regarding exposure to biomechanical factors; moreover, the vehicle trade and repair sector presented a greater risk than the reference. Indeed, in this sector, the activities are very much oriented towards uncomfortable postures, handling and carrying loads, and prolonged standing work, which makes up 80% of the working time in some subsectors such as the clothing sector [41]. Those in the human health and social work sector had a risk of reporting exposure to biological agents that was higher than that of the reference sector (administrative and support services). The risk of exposure was high, as workers in this sector of activity are very often in contact with body fluids [19]. Whereas in other sectors being in contact with biological agents seems to be an exceptional situation, this sector must deal with these biological agents. Except for the manufacturing and construction sectors, all other sectors had a lower risk of reporting exposure to chemical agents. In construction and manufacturing, the processes and raw materials used expose workers to chemical agents [42,43]. The data showed that workers in these two sectors mostly reported working with chemicals or cosmetics and being exposed to fumes, gases, and dust. Despite the provision of personal protective equipment, these can only limit and not eliminate the risk associated with exposure. This is possible as long as workers use it correctly during their activities because, in many cases, this personal protective equipment can also constrain perceptions and workers’ activities [44]. Atypical working hours appear to be a specific feature of the transport and storage, and human health and social work sectors [45]. Atypical working hours are the result of the requirements of some sectors in relation to the activities carried out. In the transport sector, atypical working hours are the result of adapting the activity in relation to the hours when the traffic flows best and to meet customers’ needs. In human health and social work, it is instead the permanent nature of the activity in this field that forces workers to declare more exposure to atypical working hours. The human health and social work sector was the sector with the most workers reporting exposure to psychosocial risk factors. This result seems to be in line with the results from the analysis of the EVREST data [39]. In part, this is explained by the high time pressure that can exist in this sector and the difficulty of reconciling private and professional life, which is consistent with the high levels of exposure to atypical hours in this sector.

### 4.3. Limitations

This study presents the first analysis between occupational exposures and activity sectors drawn from the current activity of an occupational health service and based on self-reported risk factors. This allowed us to avoid the three main biases that can occur in occupational epidemiology studies, namely selection bias, information bias, and confusion bias [46]. The profile of the workers who responded to the questionnaire and were included in this study, and the workers followed up in the occupational health service of the Cher department were very similar and were present in the same proportions in the study. Moreover, the large number of workers included in the study helped to minimize the selection bias. Through the self-reporting of exposures by workers, it was possible to have an information bias because the assessment of exposure by workers can be minimized [47,48]. This bias was mitigated because the workers do not directly report their exposures but work situations, which allowed us to deduce the inherent exposures. The self-reported questionnaire was based on the in-depth knowledge that workers have of their work environment and activity. For example, exposure to biological agents is conditioned by working in contact with such agents or working in contact with animals or their products (e.g., waste treatment). It is also important to note that most studies on occupational exposures have used self-reported data, such as the EVREST or NHANES studies [14]. Regarding the confounding bias, the groupings used for the sectors of activity were based on the activities carried out by each branch [21]. The direct relationship between the work situation and exposure was established by a detailed assessment of the workers’ work environment. The work situations were determined by questionnaires completed by the workers themselves. Given the nature of the data collection process and the close relationship between the work situation and exposure, we can conclude that the link between occupational exposures and the sectors of activity is a direct link without potential confounding factors because exposures are a derivative of only the work situations [49]. It is important to note that the study’s goal was to identify the differences in exposure between sectors and not to find causality, so the confounding factors are not a crucial point in this matter. Another point is that if any confounding factors are present, they are likely be distributed across all sectors, rather than being specific to certain sectors. Furthermore, there are studies on occupational exposures involving this type of grouping at different levels [50]. Unfortunately, the self-reported questionnaire was not designed to retrieve exposure to carcinogens. However, these work situations have been accounted for. As there may be specificities among companies in certain sectors, the results presented here are only a presentation of the overall situation observed over the study period.

## 5. Conclusions

Our study reported an overview of occupational exposures by sector alongside the sectors of activity most exposed to each exposure, using routine occupational health data. Psychosocial risk factors were commonly reported in all sectors. Workers from the construction and the human health and social work sectors seemed to report more exposures than other sectors. The analysis of occupational exposures is a necessary basis for guiding occupational health policies on the reduction of occupational exposures, the development of action plans for health and safety in enterprises, and for prevention measures involving the employer and the worker.

## Data Availability

The datasets generated during and/or analyzed during the current study are available from the corresponding author on reasonable request.

## Appendix A. Details of the Composition of Exposure Groups

Physical factors	Noise
	Working in bad weather
	Vibrating machines and tools
	Ultrasound
	Extreme temperatures (hot or cold)
	Non-ionizing radiation
	Ionizing radiation
Biomechanical factors	Awkward postures
	Handling and carrying of loads
	Repetitive motions
Biological factors	Work in contact with humans or products of human origin
	Waste treatment and disposal
	Processing in the environment or in the food industry
	Work in contact with animals or their products
Chemical agents	Handling chemicals or cosmetics
	Exposure to fumes, gases, and/or dust
	Fiber (asbestos, refractory ceramic, insulation wool, etc.)
Atypical hours	Weekend work
	Working irregular hours
	Work in alternating shifts
	Night work
	Night shifts
Psychosocial factors	Emotional demands of the job
	Work requirements
	Autonomy of organization
	Socioeconomic fears
	Support at work (colleagues, employer, supervisor)
	Recognition at work (colleagues, employer, supervisor)
	Conflict at work

## Appendix B. Details of the Activities Related to Each Sector

Administrative and support service activities	Rental and leasing activities, employment-related activities, investigation and security, building and landscape maintenance services, other support activities
Manufacturing	Manufacturing of food, beverages, tobacco-based products, textiles, and others
Construction	All construction activities
Wholesale and retail trade, repair of motor vehicles and motorbikes	Wholesale and retail trade, and repair of automobiles and motorcycles
Transport and storage	Land transportation and pipeline transportation, postal and courier activities, storage, and transportation support services
Public administration	Public administration and defense, compulsory social security
Human health and social work	Human health activities, medical and social care, and social action without accommodation

## Appendix C. Table for the Interpretation of Cramer’s V

ES > 0 denotes no or very weak association, 0.10 < ES < 0.15 denotes a moderate association, and 0.25 > ES > 0.15 denotes a strong association.

**Cramer’s V**	**Interpretation**
> 0.25	Very strong
> 0.15	Strong
> 0.10	Moderate
> 0.05	Weak
> 0	No or very weak

## Appendix D. Frequency Tables of Workers Included in the Study

**Sectors**	**n (%)**	**Men**	**Women**
Manufacturing	3145 (15.8)	994 (31.7)	2141 (68.3)
Construction	4351 (21.8)	1083 (25.7)	3135 (74.3)
Wholesale and retail trade, and repair of motor vehicles and motorbikes	1998 (10.0)	120 (6.0)	1873 (94.0)
Transport and storage	3752 (18.9)	1445 (38.7)	2293 (61.3)
Administrative and support service activities	1556 (5.8)	165 (14.5)	977 (85.6)
Public administration	2256 (11.3)	1441 (64.0)	811 (36.0)
Human health and social work	3233 (16.3)	2521 (78.0)	712 (22.0)
Total	19,711 (100)	7769 (39.4)	11,942 (60.6)
